# Dissecting the Molecular Determinants of GABA_A_ Receptors Current Rundown, a Hallmark of Refractory Human Epilepsy

**DOI:** 10.3390/brainsci11040441

**Published:** 2021-03-30

**Authors:** Pierangelo Cifelli, Silvia Di Angelantonio, Veronica Alfano, Alessandra Morano, Eleonora De Felice, Eleonora Aronica, Gabriele Ruffolo, Eleonora Palma

**Affiliations:** 1 Department of Applied Clinical and Biotechnological Sciences, University of L’Aquila, 67100 L’Aquila, Italy; pierangelo.cifelli@univaq.it; 2 Department of Physiology and Pharmacology, Istituto Pasteur-Fondazione Cenci Bolognetti, University of Rome Sapienza, 00185 Rome, Italy; silvia.diangelantonio@uniroma1.it (S.D.A.); veronica.alfano@uniroma1.it (V.A.); eleonora.df.22@gmail.com (E.D.F.); eleonora.palma@uniroma1.it (E.P.); 3 Center for Life Nanoscience, Istituto Italiano di Tecnologia (IIT), 00161 Rome, Italy; 4 Department of Human Neuroscience, University of Rome Sapienza, 00185 Rome, Italy; alessandra.morano@hotmail.it; 5 Department of (Neuro)Pathology, Amsterdam UMC, University of Amsterdam, 1105 Amsterdam, The Netherlands; e.aronica@amsterdamumc.nl; 6 Stichting Epilepsie Instellingen Nederland, 0397 Heemstede, The Netherlands

**Keywords:** GABA_A_ receptor, GABA_A_ rundown, electrophysiology, human epilepsy

## Abstract

GABA_A_ receptors-(Rs) are fundamental for the maintenance of an efficient inhibitory function in the central nervous system (CNS). Their dysfunction is associated with a wide range of CNS disorders, many of which characterized by seizures and epilepsy. Recently, an increased use-dependent desensitization due to a repetitive GABA stimulation (GABA_A_ current rundown) of GABA_A_Rs has been associated with drug-resistant temporal lobe epilepsy (TLE). Here, we aimed to investigate the molecular determinants of GABA_A_ current rundown with two different heterologous expression systems (*Xenopus* oocytes and human embryonic kidney cells; HEK) which allowed us to manipulate receptor stoichiometry and to study the GABA_A_ current rundown on different GABA_A_R configurations. To this purpose, we performed electrophysiology experiments using two-electrode voltage clamp in oocytes and confirming part of our results in HEK. We found that different degrees of GABA_A_ current rundown can be associated with the expression of different GABA_A_R β-subunits reaching the maximum current decrease when functional α1β2 receptors are expressed. Furthermore, the blockade of phosphatases can prevent the current rundown observed in α1β2 GABA_A_Rs. Since GABA_A_R represents one important therapeutic target in the treatment of human epilepsy, our results could open new perspectives on the therapeutic management of drug-resistant patients showing a GABAergic impairment.

## 1. Introduction

GABA is the main inhibitory neurotransmitter in central nervous system (CNS), able to bind several classes of receptors, namely GABA_A_ receptors (GABA_A_Rs), GABA_B_ receptors (GABA_B_Rs) and GABA_C_ receptors (GABA_C_Rs) [1]. Between them, GABA_A_Rs are responsible of both fast and tonic inhibition in CNS and represent interesting pharmacological targets for diseases characterized by dysfunctions of the inhibitory transmission. Indeed, GABAergic impairment has been reported in different pathological conditions, most of which characterized by recurrent drug-resistant seizures such as temporal lobe epilepsy (TLE) [2,3], Rett syndrome [4] and tuberous sclerosis complex (TSC) [5,6].

Specifically, GABA_A_Rs are pentameric transmembrane ionotropic, chloride-permeable receptors formed by assembling different combinations of 19 subunits [7] and different stoichiometries reflect different functional activities. This difference strongly depends on the cell type (e.g., interneurons), the subcellular localization, the brain region considered, the stage of brain development. Moreover, it has been reported that various epileptic disorders can also influence GABA_A_Rs subunit expression [5,6,7,8,9,10].

In spite of this great heterogeneity, it is well accepted that the α1β2γ2 subunit composition represents the most widespread GABA_A_R subtype in the CNS [7]. This stoichiometry predominates at postsynaptic level and mediates fast neuronal inhibition (also known as “phasic inhibition”). On the other hand, the α4-α6- and δ-containing GABA_A_Rs, also called extrasynaptic, are responsible for the slow neuronal inhibition (or “tonic inhibition”) [7,11].

Furthermore, it is well known that the binding site for GABA is located at the interface between α and β subunits, and that these latter may serve as important targets for kinases and phosphatases [1].

The failure of GABA mediated inhibitory effect is, up to now, considered one the main factors involved in seizures and epilepsy [12,13].

Among the epileptic disorders, TLE is the most frequent condition in adults associated with recurrent drug-resistant seizures [14,15], and one the most studied in both human and models.

In this framework, we previously reported both in human TLE pyramidal neurons [2] and in pilocarpine model of TLE [8], a minor responsiveness of GABA_A_Rs upon repetitive stimulation elicited by high concentrations of GABA [16] (as it occurs during epileptic seizures). This phenomenon, called GABA current rundown [2,12,16] initially appears in the limbic area (hippocampus) at the time of the first spontaneous seizures to subsequently spread to cortex during the chronic period of the disease [8,17], thus showing region- and time- dependency.

Moreover, the GABA_A_Rs current rundown is significantly reduced by phosphatase inhibitors [18], certain neurotrophic factors and chemokines [19], suggesting that an imbalance in the phosphorylation/dephosphorylation processes of GABA_A_Rs subunits and/or associated proteins may be linked to this phenomenon. Interestingly, the expression of GABA_A_-Rs subunits may change in pathological conditions such as in patients affected by TLE, that beyond to a strong current rundown, showed an up-regulation of β-subunits [18].

Notably, many other modulators of GABA_A_Rs current rundown in TLE have been recently described, such as AEDs [20,21], cytokines [22,23], phyto-cannabinoids [24,25,26], making this phenomenon an interesting therapeutic target for this kind of human epilepsy [19].

The GABA current rundown has been also described in human hypothalamic hamartomas associated with gelastic seizures [27] and recently, in one drug-resistant epileptic patient carrying a SCN1A loss-of-function mutation who has benefited from hippocampal surgery [28]. However, although the GABA_A_Rs current rundown phenomenon can be considered as one of the main actors in human drug-resistant epilepsies, its molecular determinants still need further study.

Here, we addressed this issue taking advantage of two different expression systems (mainly with *Xenopus* oocytes and to a lesser extent with HEK transfected cells) which allowed the manipulation of GABA_A_Rs stoichiometry. Our intent was to clarify which GABA_A_R subunits are prominently involved in the rundown phenomenon. Our study, to look for another missing piece in the “rundown puzzle”, may pave the way to find new therapeutic approaches to treat neurological conditions characterized by strong GABA alterations, such as TLE.

## 2. Materials and Methods

### 2.1. Oocyte Electrophysiology

The human cDNAs (α1β1, α1β2, α1β3, ratio 1:1; α1β2γ2 ratio 1:1:2) used for these experiments were kindly provided by Dr. K. Wafford. *Xenopus* oocytes were harvested and prepared as described previously [16] and then intranuclearly injected with cDNAs encoding GABA_A_Rs using a pressure microinjector (PLI-100, Warner Instruments, Holliston, MA, USA). Animal protocols were approved by the Italian Ministry of Health (authorization no. 427/2020-PR). Membrane currents were recorded 1 to 4 days after the injection using the technique of two-electrode voltage clamp. The two microelectrodes were filled with a 3M KCl solution and oocytes were placed in a 0.1 mL recording chamber, continuously perfused with oocyte ringer (OR) at 8–10 mL/min through a gravity-driven perfusion system controlled by electronic valves (BioLogic, Claix, France). Experiments were performed at room temperature (20–22 °C) [16]. OR had the following composition (mM): 82.5 NaCl; 2.5 KCl; 2.5 CaCl_2_; 1 MgCl_2_; 5 HEPES, adjusted to pH 7.4 with NaOH. Unless otherwise specified the GABA concentration was 500 μM and holding potential (V_H_) was −60 mV. The GABA current rundown was defined as the decrease of the peak current amplitude after six consecutive GABA applications (10 s) spaced out by 40 s of washout and expressed as a percentage (I_6th peak_/I_1st peak_ × 100). In a set of experiments rundown was calculated before and after the cytoplasmic injection of okadaic acid [18] (dissolved in DMSO and stored at −20 °C as 0.1 mM stock solution) at concentration of 20 nM (DMSO final dilution, 1:5000) about 8–10 min before GABA application. In the dose-response experiments, EC_50_ was calculated by fitting with a Hill equation the data relative to GABA current amplitude obtained from oocytes after GABA applications (4 s every 4 min of wash at V_H_ = −60 mV) at different concentrations, ranging from 10 nM to 1 mM.

### 2.2. Cell Culture and Transfection

Human embryonic kidney 293 (HEK) cells were grown and transfected as previously reported [29]. Briefly, cells were grown in Dulbecco’s modified Eagle’s medium (DMEM) plus 10% fetal bovine serum (Invitrogen, Carlsbad, CA, USA) and 1% penicillin/streptomycin and transiently transfected using Lipofectamine 2000 (Invitrogen), adding cDNA of each subunit per dish (α1β2 ratio 1:1; α1β2γ2 ratio 1:1:2). HEK cells were mechanically dissociated and replated onto glass coverslips 24 h before measurements and used for electrophysiological experiments 48 h after transfection.

### 2.3. Patch Clamp Recordings

During recording cells were continuously superfused at room temperature (23–26 °C) with a standard external medium containing (mM): 140 NaCl, 2.8 KCl, 2 CaCl_2_, 2 MgCl_2_, 10 Hepes, 10 glucose, pH 7.3. The patch pipettes (2–5 MΩ tip resistance) were filled with a solution containing (mM): 140 CsCl, 2 MgCl_2_, 10 Hepes- CsOH, 2 MgATP, 5 BAPTA; pH 7.3. GABA evoked currents were recorded in voltage clamp configuration using an Axopatch 200B amplifier (Molecular Devices, Sunnyvale, CA, USA), driven by pCLAMP 9 (Molecular Devices). Sampling rate was 2 kHz. Patch series resistance was compensated for by 80–90%. Cell capacitance, which ranged between 10 and 30 pF, was measured using compensation of capacitive transients. Holding potential was −70 mV and cells were continuously superfused using a gravity-driven fast exchanger perfusion system (BioLogic, Claix, France). Solution exchange time, estimated by measuring open-tip junction currents with diluted perfusion solution, ranged between 0.5 and 1.6 ms, depending on the height of solution reservoirs. After patching the cell, it was stabilized with low frequency stimulations (0.2 s GABA, 100 μM every 120 s). Afterwards, the current rundown during repetitive GABA applications (100 μM for 0.5 s every 30 s) was calculated as the peak amplitude of the sixth response as a percentage of the peak amplitude of control at first application of rundown protocol (I_6th peak_/I_1st peak_ × 100).

### 2.4. Statistical Analysis

Numbers (*n*) refer either to oocytes or cells used in each experiment and data are presented as mean ± SEM. Before data analysis, normal distribution was assessed with Shapiro-Wilk test. According to the result parametric or non-parametric tests have been used as indicated for each experiment in the figure legends. The statistical analysis of the data, including the dose-response curve fitting, was performed with Sigmaplot 12 software (Systat Software Inc, San Jose, CA, USA) and differences between two data sets were considered significant when *p* < 0.05.

## 3. Results

### 3.1. GABA Currents Evoked in Xenopus Oocytes Injected with Different Combinations of GABA_A_R Subunits

Oocytes that were not injected did not respond to any GABA application (up to 1 mM; not shown). In contrast, we successfully recorded evoked GABA currents in oocytes intranuclearly injected with cDNAs encoding different GABA_A_R subunits combinations (α1β2γ2; α1β1; α1β2; α1β3). Current evoked responses were recorded two to four days after the injection, and the application of GABA at a concentration of 500 μM (supramaximal dose) [16] determined inward currents ranging from 0.03 μA to 7.60 μA (mean: 1.22 ± 0.1 μA; *n* = 185). These currents were blocked by bicuculline (not shown) as expected and previously reported [16], thus confirming that we recorded genuine GABA currents.

**Figure 1:**
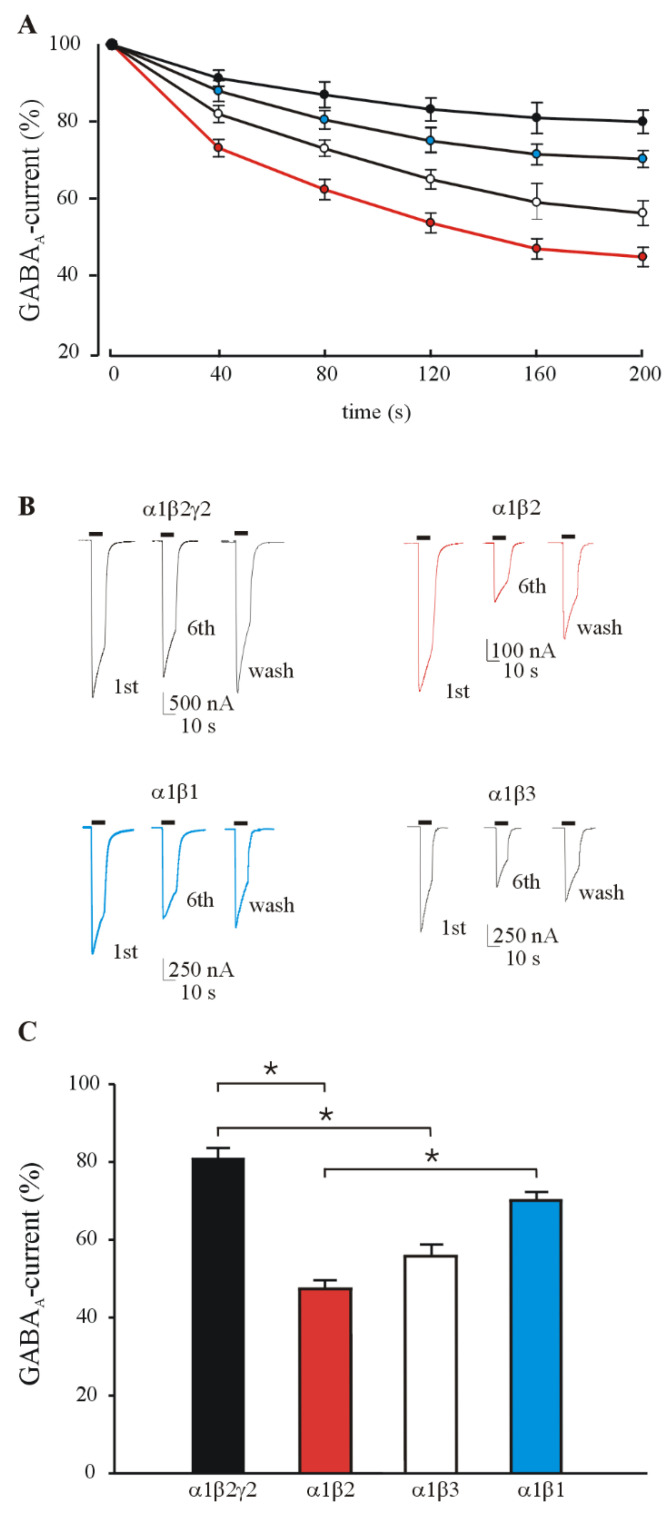
GABA current rundown in oocytes expressing different GABA_A_R subunit combinations. (**A**) Time course of GABA current rundown relative to α1β2γ2(●), α1β1(●), α1β2(●), α1β3(○) GABA_A_Rs. GABA currents are normalized to that elicited by the first GABA application (I_GABA_: ●, 2720 ± 280 nA, *n* = 24; ●, 1264 ± 106 nA, *n* = 25; ●, 749±130 nA, *n* = 95; ○, 875 ± 139 nA, *n* = 30). (**B**) Representative evoked GABA currents at first, sixth GABA application of the rundown protocol and relative washout (black bars) (**C**) The bars represent mean ± SEM of GABA current rundown values in groups of oocytes expressing different GABA receptor subunit combinations as indicated. GABA current (%) has been calculated as the reduction in peak amplitude of the 6th GABA-evoked current as percent of the 1st current after the rundown protocol (six 10-s applications of 500 μM GABA at 40-s intervals). Statistical significance among the four groups was assessed with Kruskal-Wallis One Way Analysis of Variance on ranks with pairwise multiple comparison procedures (Dunn’s Method). * *p* < 0.05.

In another set of experiments, we measured the GABA current rundown induced by repetitive GABA application (500 μM; see methods for rundown protocol) in oocytes expressing different GABA_A_R subunits combinations. As previously reported [22], the α1β2γ2 GABA_A_Rs were characterized by scarce current rundown (80.80 ± 2.8%; *n* = 24; Figure 1A,B), which is similar to that recorded from control human brain tissues [2,18] and easily recovered by washing with Ringer solution (OR, up to 4 min, see also Figure 1).

Interestingly, all the GABA_A_R combinations lacking the γ2 subunit exhibited a higher desensitization upon repetitive stimulation. Indeed, the recorded rundown for α1β2, α1β3 and α1β1 was respectively: 44.90 ± 1.80% (*n* = 95), 56.25 ± 3.28% (*n* = 30) and 70.30 ± 2.20 (*n* = 25) (Figure 1A–C) showing for the first two compositions a clear statistical significance (*p* < 0.05; see Figure 1A–C) compared to the rundown recorded in α1β2γ2 expressing oocytes. The observed percentages of current rundown was not accompanied by significant changes in the current decay (T_0.5_ = 12.04 ± 0.80 s, 1st GABA application and 11.47 ± 0.45 s, 6th GABA application for α1β2γ2; 11.13 ± 0.34 s, 1st GABA application and 11.08 ± 0.32 s 6th GABA application for α1β2).

### 3.2. GABA Current Rundown in HEK Transfected Cells

In order to confirm the data obtained in the oocytes using α1β2γ2 and α1β2 GABA_A_R composition, we decided to transfect only these cDNAs in HEK cell line to perform whole-cell patch-clamp recordings [29].

**Figure 2:**
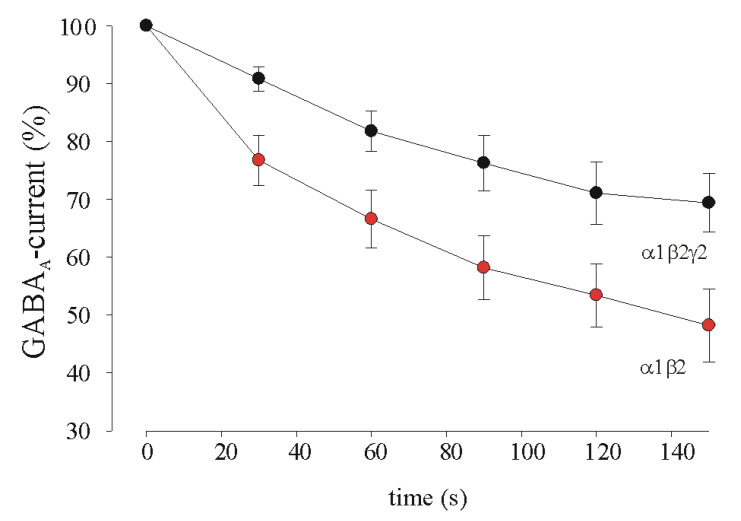
GABA current rundown in HEK cells transfected with GABA_A_Rs cDNAs. Time course of averaged responses (±SEM; *p* < 0.05, Mann–Whitney rank sum test) obtained in HEK cells expressing either α1β2γ2 (*n* = 11, ●) or α1β2 (*n* = 13, ●) GABA_A_Rs. In each cell, peak amplitude of subsequent responses was expressed as a percentage of the first response of the rundown protocol.

GABA (100 μM), applied via rapid solution exchanger positioned above HEK cells, elicited a rapid inward current. After obtaining stable inward currents with low-frequency (0.5 s every 2 min) applications, repetitive GABA applications (0.5 s every 30 s, see also methods and [2], Figure 2) caused a progressive decrease in current amplitude in both α1β2 and α1β2γ2 receptor expressing cells, with a recovery towards initial values when agonist was applied at longer time intervals (120 s). Notably, while in cells expressing α1β2γ2 GABA current rundown was 69 ± 5% (*n* = 11, *p* < 0.05 respect to α1β2), the cells lacking γ2 subunit, showed a GABA current rundown of 48 ± 6% (*n* = 13) thus confirming the data obtained in injected oocytes for α1β2 composition.

### 3.3. Phosphatase Inhibition Recovers GABA Current Rundown

**Figure 3:**
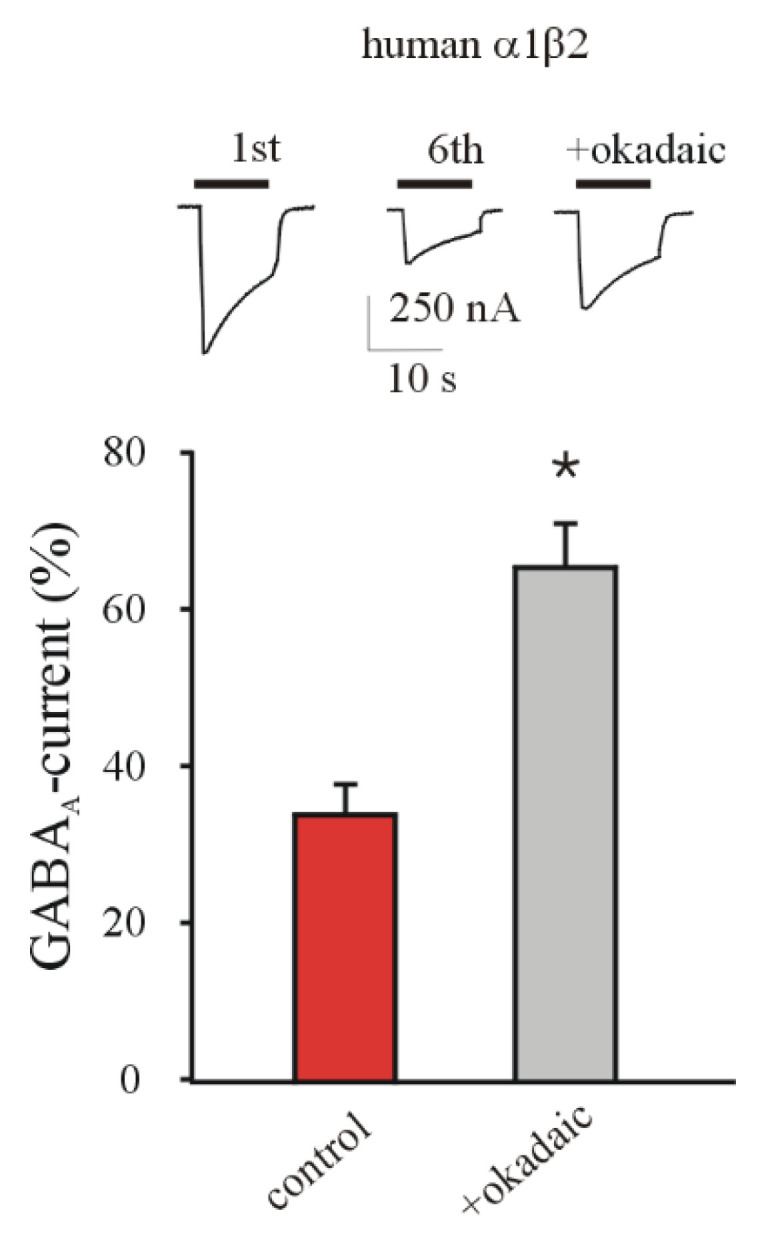
Effect of okadaic acid on GABA current rundown in oocytes expressing α1β2 GABA_A_Rs. The bars represent mean ± SEM of GABA current rundown values before (red bar) and after (grey bar) the intracellular injection of okadaic acid (≈20 nM final concentration) *n* = 11; * *p* < 0.001, Wilcoxon Signed Rank Test. Inset: representative currents in one oocyte at first and sixth GABA application of rundown protocol and relative recovery after application of okadaic acid.

Previous experiments on epileptic human tissues demonstrated that okadaic acid, a phosphatase inhibitor, is able to bring back the GABA current rundown to more physiological levels [2,18,27], close to those recorded in control tissues. Here, our experiments showed that okadaic acid (8–10 min pretreatment at ≈20 nM final concentration before GABA application) recovered the GABA current rundown in oocytes expressing α1β2 GABA_A_Rs (34.06 ± 4.34% versus 65.78 ± 5.64%, respectively before and after okadaic acid treatment, *n* = 11; Figure 3; *p* < 0.001) without affecting GABA current amplitudes (I_GABA_: 625 ± 170 nA and 595 ± 195 nA, first GABA application before and after okadaic acid).

### 3.4. α1β2γ2 and α1β2 GABA_A_Rs Display Similar GABA Sensitivity in Xenopus Oocytes

**Figure 4:**
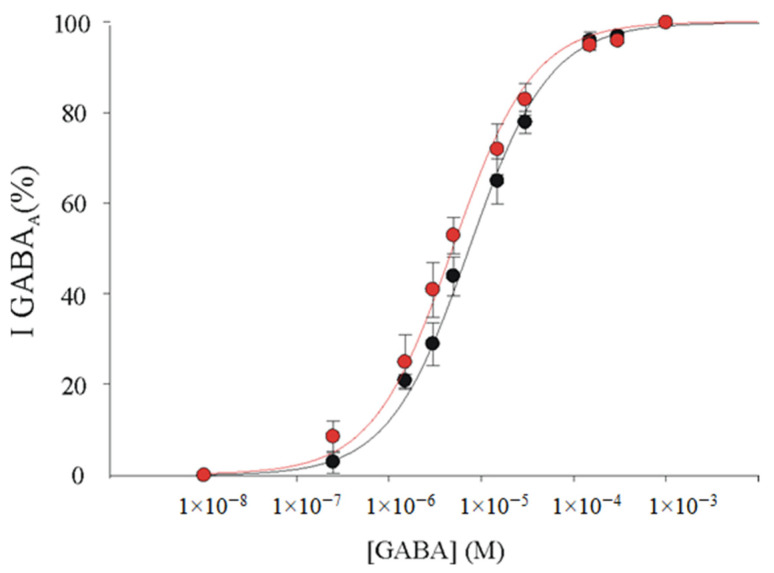
GABA_A_Rs affinity in oocytes expressing α1β2γ2 GABA_A_Rs or α1β2 GABA_A_Rs. GABA dose–current relationships in oocytes injected with α1β2γ2 (●) or α1β2 cDNAs (●). Peak currents were normalized to the current obtained with 1 mM GABA and refer to 6 oocytes each point. EC_50_ and n_H_ were 7.36 ± 0.47 μM and 1.0 ± 0.04 for α1β2 receptors; 5.0 ± 0.15 μM and 0.99 ± 0.1 for α1β2γ2; *p* > 0.05, Mann–Whitney rank sum test.

In order to investigate if the difference in GABA rundown was due to a difference in GABA sensitivity (e.g., an increase of GABA sensitivity in α1β2 receptors could explain an increased desensitization), further experiments were performed to evaluate this parameter for the α1β2γ2 and α1β2 subunit compositions. To this purpose, we constructed dose-response relationships using oocytes expressing the α1β2γ2 or α1β2 GABA_A_Rs. In our experiments, we did not find any difference in GABA sensitivity between α1β2γ2 receptors (EC_50_ = 7.36 ± 0.47 μM, Hill number (n_H_) = 1.0 ± 0.04; *n* = 6; Figure 4) and α1β2 receptors (EC_50_ = 5.0 ± 0.15 μM, n_H_ = 0.99 ± 0.1; *n* = 6; Figure 4; *p* > 0.05).

## 4. Discussion

The refractoriness to the currently available treatments is still a prominent problem in the management of drug-resistant epileptic patients and thus the need of new therapeutic targets is increasingly urgent [30]. GABA current rundown has been characterized in TLE patients as a hallmark of GABAergic impairment [2] and previous experiments have provided insight on how this phenomenon might be caused by a sort of “instability” of GABA_A_Rs due to an alteration of receptor phosphorylation [8,18,22,31]. This “instability” could be detrimental during seizures when high concentrations of GABA are released at high frequency inducing a decrease of its inhibitory efficacy.

Even if GABA current rundown was described and characterized in different tissues, such as TLE brain slices [2] and epileptic hypothalamic hamartomas [27], the molecular determinants of the aforementioned instability are not yet completely understood.

Here, we performed further investigation to unravel these still unknown aspects of GABA current rundown, and we found that: (i) γ-lacking GABA_A_Rs expressed in *Xenopus* oocytes are characterized by an increased current rundown; (ii) among the γ-lacking GABA receptors, the α1β2 GABA_A_Rs exhibit the strongest rundown; (iii) these latter results were also demonstrated in HEK cells expressing human α1β2 GABA_A_Rs; (iv) okadaic acid, a phosphatase inhibitor, was able to decrease GABA current rundown in oocytes expressing α1β2 GABA_A_Rs; (v) the observed differences cannot be attributed to changes of the affinity for GABA or current decay between α1β2γ2 and α1β2 receptor isoforms. This last finding is in line with previous evidence showing that agents ameliorating GABA current rundown through PKC-mediated phosphorylation did not affect current desensitization [21,31]. The finding of an increased current rundown in γ-lacking GABA_A_Rs where β-subunits are predominant (α1β2, α1β3 and α1β1 GABA_A_Rs) is particularly interesting because it is well-known that both γ- and β-subunits possess key phosphorylation sites which are involved in the modulation of GABA_A_Rs function [32,33].

Two key conclusions stem from these results: first, that in the most common GABA_A_R isoform in CNS (α1β2γ2) [7,34] the presence of γ–subunits possess an important role in receptor stabilization [33,35] and, second, the various β-subunits may differently contribute to the mechanisms of GABA current rundown, because the effect of receptor phosphorylation may vary depending upon the involved β-subunit [36,37].

The difference between α1β2γ2 and α1β2 GABA_A_R rundown was also found in HEK cells. This is not only important as a confirmation of our results, but it also excludes the contribution of the host cell biosynthetic machinery to the reported phenomena. In addition, these experiments also ruled out that endogenous membrane proteins in both the expression systems may have influenced our results.

Therefore, thanks to this set of experiments in HEK cells we can conclude that the GABA current decrease upon repetitive GABA stimulation is similar, at least in this regard, between *Xenopus* oocytes and transfected human cells. This is not surprising since we have previously shown that the main electrophysiological parameters are well conserved in the two expression systems [38].

These findings acquire additional value in the light of understanding how GABA_A_R receptor rundown arises in the brain of epileptic drug-resistant patients [2].

As it was mentioned, TLE can cause a dysregulation of GABA_A_R subunit composition. This alteration has been well-characterized both in animal models of epilepsy [39] and in human TLE patients [40,41].

Altogether, these results suggest that GABA_A_Rs function may be influenced by: (i) a different assembly of the receptor itself, because of the modification of the relative expression of its subunits and (ii) the modulation of the receptor function by mechanisms such as phosphorylation, to which a “pathologic” receptor may become more subject to. Further experiments will better elucidate these specific points.

Notably, several previous reports demonstrated that there is a constant modulation of the β-subunit mRNA expression in TLE [18,42]. This is in line with our present results and suggests that the specific targeting of GABA_A_ subunits phosphorylation sites may be a promising strategy to promote the recovery of the inhibitory function in TLE.

To support this point, previous experiments already shown that blocking the phosphatases allowed to restore a more physiological GABA current rundown in TLE brain tissues [2,18] and human epileptic hamartomas [27]. Here, we tested the positive effect of okadaic acid (a broad-spectrum phosphatase inhibitor) also on GABA current rundown of GABA_A_Rs expressed in oocytes. Since the receptors expressed in this system are not accompanied by other exogenous proteins, these experiments suggest that the phosphatase action is specifically targeted on GABA_A_Rs subunits.

It is well-known that current desensitization may be influenced by modifications of the receptor’s affinity for neurotransmitter. In the light of our results, it is unlikely that the highest degree of GABA current rundown in α1β2 GABA_A_Rs is due to an increase of GABA sensitivity, because we did not find a different EC_50_ nor a faster current decay in α1β2 versus α1β2γ2 GABA_A_Rs.

Further studies will be needed to better clarify the exact cellular mechanism of the rundown phenomenon and the events induced by the unbalance of phosphorylation/dephosphorylation state of the GABA_A_Rs.

Nonetheless, we demonstrated for the first time, using two different expression systems, that GABA current rundown strongly depends on the GABA_A_R subunit composition and that different β–subunits correlate with different degrees of rundown. Finally, given the importance of GABA_A_R rearrangement in TLE [43], this study may pave the way to new specific agents selectively able to ameliorate the GABAergic impairment in different epileptic disorders.

## 5. Conclusions

We found that a use-dependent GABAergic impairment (i.e., GABA rundown) which is considered a hallmark of human epileptic diseases, can be mimicked by expressing GABA_A_Rs containing different β-subunits reaching a value similar to epileptic human tissues for α1β2 combination. This rundown was ameliorated by blocking the phosphatases confirming that this phenomenon is associated with an unbalance of phosphorylation/dephosphorylation mechanism leading to a sort of “instability” of GABA_A_Rs. Since the beta subunits that are up-regulated in human epileptic tissues, contain phosphorylation sites, we suggest that agents acting on phosphorylation pathways could mitigate the GABAergic impairment described in drug-resistant epileptic patients. Further experiments will better elucidate this point.
